# Axonal chronic injury in treatment-naïve HIV+ adults with asymptomatic neurocognitive impairment and its relationship with clinical variables and cognitive status

**DOI:** 10.1186/s12883-018-1069-5

**Published:** 2018-05-10

**Authors:** Rui-li Li, Jun Sun, Zhen-chao Tang, Jing-ji Zhang, Hong-jun Li

**Affiliations:** 1 Department of Radiology, Beijing YouAn Hospital, Capital Medical University, No.8, Xi Tou Tiao, Youanmen Wai, Feng Tai District, Beijing, 100069 China; 2 School of Mechanical, Electrical & Information Engineering, Shandong University, No.180, West Wenhua Road, Weihai, 264209 Shandong Province China; 3 STD and AIDS clinical treatment center, Beijing YouAn Hospital, Capital Medical University, No.8, Xi Tou Tiao, Youanmen Wai, Feng Tai District, Beijing, 100069 China

**Keywords:** HIV, Asymptomatic neurocognitive impairment, White matter, Diffusion tensor imaging

## Abstract

**Background:**

HIV is a neurotropic virus, and it can bring about neurodegeneration and may even result in cognitive impairments. The precise mechanism of HIV-associated white matter (WM) injury is unknown. The effects of multiple clinical contributors on WM impairments and the relationship between the WM alterations and cognitive performance merit further investigation.

**Methods:**

Diffusion tensor imaging (DTI) was performed in 20 antiretroviral-naïve HIV-positive asymptomatic neurocognitive impairment (ANI) adults and 20 healthy volunteers. Whole-brain analysis of DTI metrics between groups was conducted by employing tract-based spatial statistics (TBSS), including fractional anisotropy (FA), mean diffusivity (MD), axial diffusivity (AD) and radial diffusivity (RD). DTI parameters were correlated with clinical variables (age, CD4^+^ cell count, CD4^+^/CD8^+^ ratio, plasma viral load and duration of HIV infection) and multiple cognitive tests by using multilinear regression analyses.

**Results:**

DTI quantified diffusion alterations in the corpus callosum and corona radiata (MD increased significantly, *P* < 0.05) and chronic axonal injury in the corpus callosum, corona radiata, internal capsule, external capsule, posterior thalamic radiation, sagittal stratum, and superior longitudinal fasciculus (AD increased significantly, *P* < 0.05). The impairments in the corona radiata had significant correlations with the current CD4^+^/CD8^+^ ratios. Increased MD or AD values in multiple white matter structures showed significant associations with many cognitive domain tests.

**Conclusions:**

WM impairments are present in neurologically asymptomatic HIV+ adults, periventricular WM (corpus callosum and corona radiata) are preferential occult injuries, which is associated with axonal chronic damage rather than demyelination. Axonopathy may exist before myelin injury. DTI-TBSS is helpful to explore the WM microstructure abnormalities and provide a new perspective for the investigation of the pathomechanism of HIV-associated WM injury.

## Background

HIV can enter the central nervous system (CNS) soon after seroconversion and cause persistent CNS inflammation [1]. With ongoing injury to the brain, it may lead to cognitive, behavioural and motor abnormalities, which are called HIV-associated neurocognitive disorders (HAND) [2]. HAND can be clinically subdivided into three categories: asymptomatic neurocognitive impairment (ANI), mild neurocognitive disorder (MND) and HIV-associated dementia (HAD) [3]. ANI is the mildest and most common type of HAND (accounting for 70%), which is characterized by mild cognitive impairment on neuropsychological performance tests without obvious accompanying difficulties in daily functioning [4]. HAD is the most severe form. These definitions are mainly based upon an individual’s performance on multiple cognitive domains and a brief self-report of cognitive difficulties in daily life. These neuropsychological tests are time consuming (~ 3 h) and are usually performed in specific research institutions [3], so patients in ANI stages are rarely diagnosed in traditional outpatient visits (15–30 min).

Conventional structural magnetic resonance imaging (MRI) scans are unable to detect early HIV-associated brain white matter (WM) abnormalities [5]. As a noninvasive and rapidly evolving MRI technique, diffusion tensor imaging (DTI) can measure the diffusion of water molecules in WM, and more recently, it has become a popular method for studying HIV-induced WM microstructural integrity [6–12]. So far, tract-based spatial statistics (TBSS) is the most frequently recommended and employed method of analysis for DTI [13], which overcomes the limitations of conventional methods (region of interest, ROI; voxel-based analysis, VBA). There are four parameters for DTI, including fractional anisotropy (FA), mean diffusivity (MD), axial diffusivity (AD), and radial diffusivity (RD). As a marker of the diffusion directionality of water molecules, FA can reflect the deviation of water motion and provide information about the microstructural integrity of highly oriented microstructures [14]. MD is a marker of the molecular motion speed and can reflect the average diffusion in all three directions [15]. AD is assumed to reflect diffusivity parallel to the WM tract, and RD represents diffusion perpendicular to the tract [16]. Generally, FA and MD are influenced by AD and RD. Decreased FA and increased MD are measures of neuronal injury, increased AD is a measure of axonal chronic damage, and increased RD is a measure of myelin damage [7, 17].

Though many DTI studies on HIV have reported a loss of WM integrity [6–12], few studies have focused on the WM microstructure in neuroasymptomatic HIV+ individuals without treatment. Zhu T et al. found that WM injuries in neurologically asymptomatic HIV patients are mainly located in the posterior part of both hemispheres (MD, AD, RD increased significantly) [7]. Wang B et al. observed a decrease in FA in the corpus callosum and anterior corona radiata and an increase in MD, RD, and AD in most skeleton locations [8]. In both studies, the neuropsychological tests were assessed by AIDS Dementia Complex (ADC) staging according to the Memorial Sloan Kettering (MSK) staging system, and an ADC score of 0 or 0.5 was considered neurocognitive asymptomatic. Zhuang Y et al. investigated WM changes in ANI patients diagnosed according to the Frascati criteria [3] and found no significant WM microstructural differences between HIV-infected and healthy controls [18]. Cysique LA et al. reported that the location of WM injury in ANI cases was the anterior limb of the internal capsule [19]. The above findings still exhibit differences.

HIV-associated WM damage includes demyelination and axonal injury; however, the relationship between them and the neuropathology of HIV-related WM impairment is still unclear. The primary oligodendrocyte and myelin damage leading to secondary axonal damage (outside-in) or primary axonopathy triggering oligodendrocyte injury and demyelination (inside-out) are indistinguishable [20]. WM alterations may contribute to cognitive deficits in HIV-infected patients [7, 18, 21]. It is worth noting that the relationship between WM abnormalities and cognitive status has not been well characterized or systematically assessed.

Moreover, the effects of multiple clinical contributors on cerebral WM integrity merit further investigation. The various potential clinical influencing variables include factors that are directly related to HIV disease (i.e., CD4^+^ level, CD4^+^/CD8^+^ ratio, plasma viral load and duration of HIV infection) and factors that can affect the CNS, such as ageing. Recent studies have identified that HIV duration was significantly correlated with DTI parameters [7, 22]. FA values in the corpus callosum were negatively correlated with the duration of infection in antiretroviral-naïve primary HIV infection patients [23]. Cohen RA et al. reported that the CD4 nadir and the duration of HIV infection may be risk factors for cerebral injury [24]. However, other studies showed dissenting results that age can exacerbate HIV-associated WM abnormalities [11, 25]. Regrettably, there were discrepancies in previous findings.

In the present study, we aimed to investigate the WM microstructural changes in treatment-naïve HIV patients with ANI through DTI-TBSS technology. In particular, the current study was confined to treatment-naïve patients to rule out the possibility of antiretroviral therapy (ART) erosion on WM integrity. We also wanted to quantify the relationships between WM damage and age, CD4^+^ counts, CD4^+^/CD8^+^ ratio, plasma viral load, duration of HIV infection and cognitive status.

## Methods

### Subjects

The protocol was approved by the ethics committee. HIV participants were recruited from infectious disease outpatient clinic of Beijing YouAn Hospital, Capital Medical University. The inclusion criteria for patients were as follows: age ≥ 18 years, naïve to ART prior to enrolment, and HAND stage of ANI. According to the inclusion criteria, we enrolled twenty patients from June 2014 to July 2016. Twenty seronegative healthy volunteers matched for age, gender and education level were recruited from the same community by advertisements. All subjects provided written informed consent prior to enrolment. The exclusion criteria for both HIV-infected and HIV-negative participants were as follows: 1) age < 18 years; 2) neurological disorders: epilepsy, stroke, an active or known past opportunistic infection of the CNS; 3) alcohol or drug abuse within the last 6 months; 4) stable anxiety or depression, including those managed by stable anti-anxiety or antidepressant therapy; 5) contraindication to MR; 6) trauma, tumours, infection (except HIV), vascular diseases and other visible brain lesions on standard MRI (T_1_WI and T_2_-fluid attenuated inversion recovery (FLAIR)).

In the HIV-infected individuals, HIV was confirmed by an enzyme-linked immunosorbent assay and western blot analysis. The duration of HIV infection was determined according to patients’ self-reports on their risk behaviours. The recent CD4^+^ counts were performed within 2 weeks of neuroimaging. HIV RNA levels were measured from blood plasma. The mode of HIV infection was sexual contact (male homosexual contact for 14 patients, heterosexual contact for 6 patients). The years of education ranged from 13 to 19 years (mean: 16.5 ± 1.8 years).

Two to three hours prior to the MRI scanning, each patient underwent a comprehensive neuropsychological assessment, including 6 cognitive domains and a report of cognitive difficulties in daily life. Self-questionnaires of daily functioning were assessed with a short Activity of Daily Living scale [26]. The neurocognitive evaluation surveys the following abilities: verbal fluency (Animal Verbal Fluency Test, AFT), attention/working memory (Continuous Performance Test-Identical Pair, CPT-IP; Wechsler Memory Scale, WMS-III; Paced Auditory Serial Addition Test, PASAT), executive function (Wisconsin Card Sorting Tests, WCST-64), memory (learning and delayed recall) (Hopking Verbal Learning Test, HVLT-R; Brief Visuospatial Memory Test, BVMT-R), speed of information processing (Trail Marking Test A, TMT- A) and fine motor skills (Grooved Pegboard, dominant and non-dominant Hands) [4, 27]. Raw scores for each test were transformed into T-scores and adjusted for age, gender, and education level. T-scores across more tests for one cognitive domain were averaged to calculate domain-specific T-scores. Patients whose cognitive impairment involved two or more cognitive abilities (performance of at least one standard deviation below the mean for norms on neuropsychological tests) and presented no cognitive difficulties in everyday life were diagnosed with ANI [3]. All patients were diagnosed with ANI according to the Frascati criteria [3].

Twenty patients and twenty healthy controls received MRIs. All scans were reviewed by an experienced neuroradiologist for motion artefacts and evidence of unknown brain lesion, which could have affected DTI indices. The image quality of one patient was poor. After communicating with the patient, we immediately reacquired data and obtained good image quality. No MRI scans were required to be excluded from DTI analysis. Thus, we presented data for 20 patients and 20 controls.

### MRI protocols

All MRI scans were performed on a Siemens Trio 3.0 Tesla imager. Standard structural images were acquired using axial T_1_WI (repetition time (TR) = 250 ms, echo time (TE) = 2.46 ms) and T_2_-FLAIR combined fat saturation (TR = 8000 ms, TE = 2370.9 ms, inversion time = 97 ms) sequences to check whether there were visible intracranial lesions. For DTI data, a single-shot echo-planar imaging sequence was used for acquisition. The parameters for DTI were: TR = 3300 ms, TE = 90 ms, slice thickness = 4 mm with 1.2 mm gap, number of slices = 63, matrix size = 128 × 128, field of view = 230 × 230 mm, number of excitations = 3, space resolution = 1.8 mm × 1.8 mm × 1.8 mm, total acquisition time = 3.39 min. Diffusion sensitizing gradients were applied along 20 non-collinear directions with b = 1000 s/mm^2^, and one b = 0 s/mm^2^.

DTI datasets were performed and analysed using FSL5.0 (FMRIB Image Analysis Group, Oxford, UK, http://www.fmrib.ox.ac.uk/fsl) [28]. Details of the DTI processing steps, including pre-processing and TBSS processing, have been described previously [8, 29]. There were three steps for pre-processing. The raw DTI images were first corrected for the effects of eddy currents and head movements and deformations using eddy current correction within FDT. Then, brain mask extraction was performed on one of the no-diffusion-weighting (b = 0) images by running the Brain Extraction Tool in FSL. Finally, the diffusion tensor model was computed using DTIFIT within FDT for whole brain volumes to generate tensor-derived maps, including FA, MD, AD, and RD. TBSS-processing includes four steps. The first is image registration. Using the FA map as a target template for registration, more accurate results can be achieved, as FA is a normalized measure of eigenvalue standard deviation and represents the degree of diffusion directionality. A common registration target brain image template (FMRIB58_FA) was identified, and all subjects’ FA images were aligned to this target using FMRIB’s non-linear image registration tool, through which all the FA volumes were aligned to a 1.0 × 1.0 × 1.0 mm^3^ Montreal Neurological Institute standard space. Second, the mean of all aligned FA images was skeletonized, and a mean FA skeleton image (threshold = 0.2) was generated. Third, the aligned FA image for each subject was projected onto the mean FA skeleton by filling the skeleton with maximum FA values from the nearest relevant tract centre to generate a skeletonized FA map. Corresponding skeletonized maps for the other diffusion measures (MD, AD and RD) were also similarly generated. Lastly, voxelwise statistical analyses of DTI metrics were carried out on the skeleton space.

### Statistical analysis

Demographic characteristics of the HIV+ participants and healthy controls were analysed with IBM SPSS Statistics (version 22.0). Chi-squared analysis was used to evaluate the sex distribution between HIV+ patients and healthy controls. Independent t-test analysis was used to calculate the differences in age and education level between the two groups. Significance was defined as *p* < 0.05.

For TBSS analysis, voxel-wised statistics of the DTI parameters (FA, MD, AD, RD) for the two group differences were tested in the general linear model framework using the FSL randomize tool with a non-parametric permutation testing (5000 random permutations) [30]. The threshold-free cluster enhancement (TFCE) method with a threshold set at 0.95 was used to obtain correction for multiple comparisons [31], and statistical maps were obtained with family-wise error (FWE) correction at the *p* < 0.05 level. The significant group differences in tracts were located with the Johns Hopkins University (JHU)-ICBM-DTI-81 WM Label Atlas.

To investigate the relationships between DTI metrics and clinical variables and cognitive performance for HIV-positive patients, multiple linear regression analysis between DTI indices and age, CD4^+^ counts, CD4^+^/CD8^+^ ratio, plasma viral load, duration of HIV infection and scores of cognitive performance was performed. A significance level of 0.05 was obtained using IBM SPSS Statistics (version 22.0).

## Results

### Demographic information

The demographic and clinical information for HIV+ patients and healthy controls are listed in Table 1. There were no significant differences in age, sex, or education level (in years) between the HIV+ patients and healthy controls.

**Table 1:** Clinical and demographic data of study participants

Items	Patient group (*N* = 20)	Control Group (N = 20)	*p*-value
Age	30.6 ± 9.6	31.5 ± 7.6	0.325^b^
Sex (M/F)	19:1	19:1	1.000^a^
Education level (year)	16.5 ± 1.8	16.1 ± 0.8	0.372^b^
Duration of infection (year)	3.1 ± 0.9	N/A	N/A
CD4 (cells/ml)	254.6 ± 168.8	N/A	N/A
Viral load (log) (copies/ml)	4.26 ± 1.1	N/A	N/A

*N* number of subjects*, M* male*, F* female*, N/A* not applicable or available*, a* Chi-squared analysis*, b* Independent t test*,* Significance level *P* < 0.05

### White matter abnormalities in ART-naïve HIV+ patients at ANI stage

**Fig. 1:**
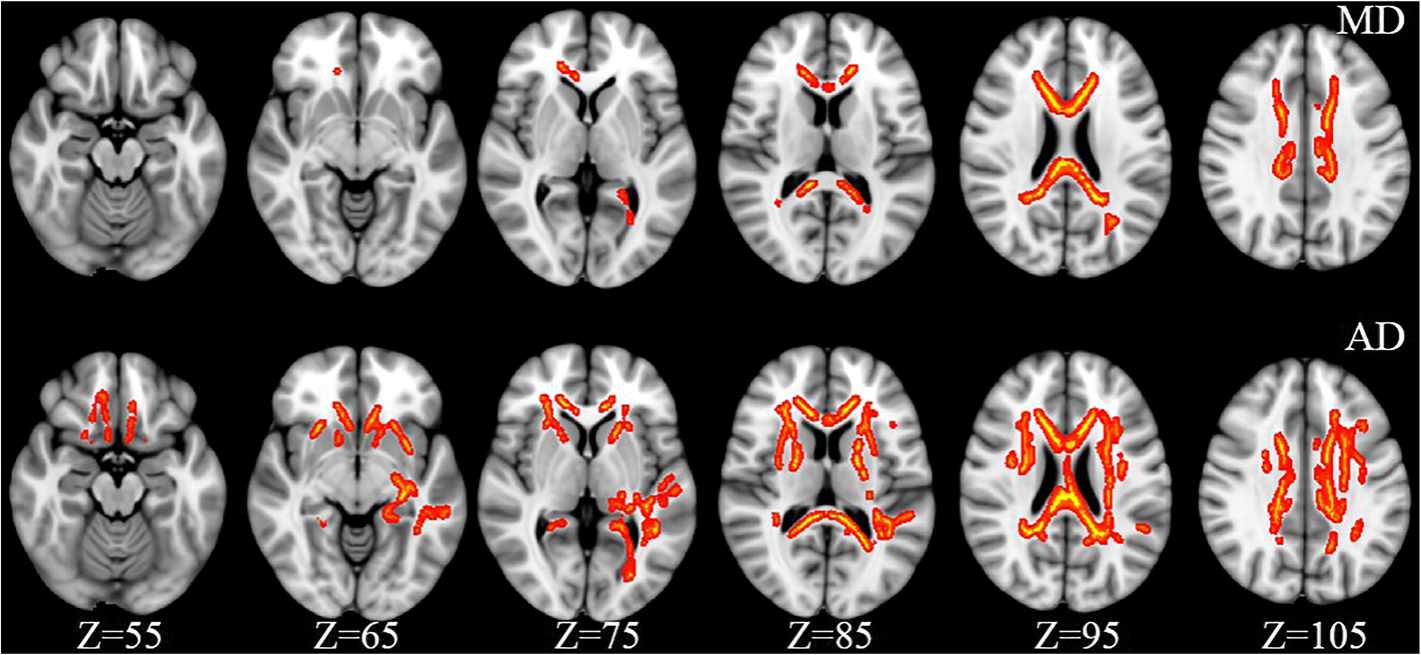
TBSS analysis of DTI indices between HIV+ and control groups (Transverse section). Areas in red-yellow are regions where MD and AD were significantly increased (*P* < 0.05, corrected by TFCE) in HIV-infected individuals compared with controls. The number below each brain image indicates the Z coordinate in the Montreal Neurological Institute (MNI) space. MD, mean diffusivity; AD, axial diffusivity

Voxel-based TBSS demonstrated significant differences in DTI parameters (MD and AD values) of HIV-infected individuals compared to controls. The FA map and RD map revealed no significant differences between the two groups. The results were illustrated in Fig. 1 and Table 2. Compared with healthy controls, HIV-positive patients exhibited significantly higher MD in the genu, body and splenium of corpus callosum, bilateral anterior and superior corona radiate. Increased AD was observed in extensive brain regions, including the genu, body and splenium of the corpus callosum; bilateral anterior and superior corona radiata, anterior limb of the internal capsule, external capsule; left retrolenticular part of the internal capsule, posterior corona radiata, posterior thalamic radiation, sagittal stratum, superior longitudinal fasciculus (all *P* < 0.05). Regions of increased AD were much more prevalent than those of MD.

**Table 2:** Location and cluster size of abnormal WM tracts between HIV patients and healthy controls

WM structures (JHU-WM Atlas)	Side	HIV patients vs. controls cluster size	
		MD	AD
Genu of corpus callosum	–	587	973
Body of corpus callosum	–	1299	1901
Splenium of corpus callosum	–	962	1615
Anterior corona radiata	R	256	918
Anterior corona radiata	L	236	716
Superior corona radiata	R	121	448
Superior corona radiata	L	255	845
Anterior limb of internal capsule	R	–	306
Anterior limb of internal capsule	L	–	363
External capsule	R	–	186
External capsule	L	–	279
Retrolenticular part of internal capsule	L	–	215
Posterior corona radiata	L	–	332
Posterior thalamic radiation	L	–	287
Sagittal stratum	L	–	105
Superior longitudinal fasciculus	L	–	358

*WM* white matter, *MD* mean diffusivity, *AD* axial diffusivity, *L* left, *R* right, *JHU-WM Atlas* the ICBM-DTI-81 White Matter Atlas

### Correlations between DTI metrics and clinical variables for HIV-infected patients

**Fig. 2:**
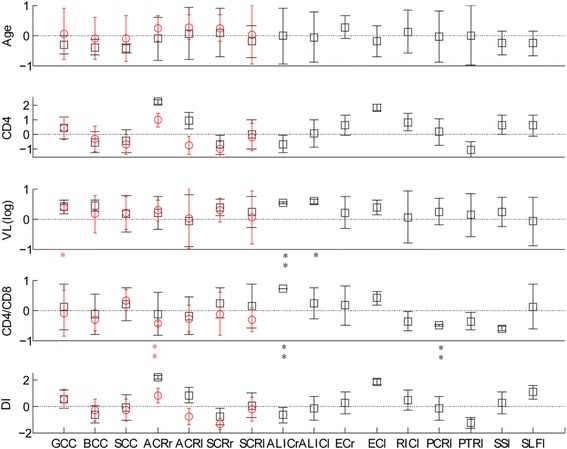
Regression coefficients and significance for clinical variables and white matter impairments. The increased MD values in the right ACR negatively correlated with CD4^+^/CD8^+^ ratios. The increased AD values in the left PCR negatively correlated with CD4^+^/CD8^+^ ratios. The increased AD values in the right ALIC positively correlated with viral load and CD4^+^/CD8^+^ ratios Note: bar: *P* value; *: *p* < 0.1; **: *p* < 0.05; red circle: regression coefficients of MD; black square: regression coefficients of AD. MD, mean diffusivity; AD, axial diffusivity; GCC, genu of corpus callosum; BCC, body of corpus callosum; SCC, splenium of corpus callosum; ACR, anterior corona radiata; SCR, superior corona radiata; ALIC, anterior limb of internal capsule; EC, external capsule; RIC, retrolenticular part of internal capsule; PCR, posterior corona radiata; PTR, posterior thalamic radiation; SS, sagittal stratum; SLF, superior longitudinal fasciculus. l, left; r, right; VL, viral load; DI, duration of infection.

Fig. 2 shows the regression coefficients and significance for clinical clinics on MD and AD values in the regions of white matter impairment. The increased MD values in the right anterior corona radiate were negatively correlated with CD4^+^/CD8^+^ ratios (*r* = − 0.437, *P* = 0.05) (Table 3). Similar analyses showed that the increased AD values in the left posterior corona radiata were negatively correlated with CD4^+^/CD8^+^ ratios (*r* = − 0.488, *P* = 0.029) (Table 4). The increased AD values in the right anterior limb of the internal capsule were positively correlated with viral load (*r* = − 0.848, *P* = 0.019) and CD4^+^/CD8^+^ ratios (*r* = − 0.717, *P* = 0.003) (Table 4).

**Table 3:** Regression coefficients and significance for MD values and clinical variables and cognitive status scores

		MD values of HIV-associated white matter impairment						
		GCC	BCC	SCC	ACR_R	ACR_L	SCR_R	SCR_L
Clinical Variables	Age	0.057	− 0.118	−0.096	0.221	0.26	0.239	0.016
	CD4	0.433	−0.282	− 0.637	0.986	− 0.724	−1.001	− 0.142
	CD4/CD8	− 0.101	− 0.331	0.331	− 0.437 ^**^	− 0.274	− 0.127	− 0.307
	VL(log)	0.381^*^	0.162	0.207	0.277	0.006	0.287	0.055
	DI	0.564	−0.281	−0.326	0.815	−0.8	−1.319	− 0.224
Cognitive Status	VF	−0.053	0.012	−0.169	−0.114	− 0.135	−0.293	− 0.459^*^
	A/WM	0.021	−0.004	−0.238	0.21	−0.001	0.424	0.141
	EF	−0.272	−0.226	− 0.2	−0.172	− 0.133	−0.194	− 0.218
	M(LDR)	−0.417^**^	−0.338	0.159	−0.511^**^	− 0.559^**^	−0.501^**^	− 0.281
	SIP	0.684^**^	0.427^*^	0.155	0.559^**^	0.583^**^	0.444^**^	0.239
	FM	−0.417^**^	−0.259	−0.403^*^	− 0.391^**^	−0.321^*^	− 0.474^**^	−0.443^*^

The regression coefficients and significance results were calculated by the multiple linear analysis method*. MD* mean diffusivity, *GCC* genu of corpus callosum, *BCC* body of corpus callosum, *SCC* splenium of corpus callosum, *ACR* anterior corona radiata, *SCR* superior corona radiata, *L* left, *R* right, *VL* viral load, *DI* duration of infection, *VF* Verbal Fluency, *A/WM* Attention/Working Memory, *EF* Executive Functioning, *SIP* Speed of Information Processing, *MS* Motor Skills, * P < 0.1, ** P < 0.05

**Table 4:** Regression coefficients and significance for AD values and clinical variables and cognitive status scores

		AD values of HIV-infected in different brain regions															
		GCC	BCC	SCC	ACR_R	ACR_L	SCR_R	SCR_L	ALIC_R	ALIC_L	EC_R	EC_L	RIC_L	PCR_L	PTR_L	SS_L	SLF_L
Clinical Variables	Age	−0.321	−0.395	−0.446	−0.111	0.053	0.078	−0.202	−0.025	− 0.065	0.27	− 0.19	0.115	− 0.051	−0.006	− 0.251	−0.264
	CD4	0.475	−0.521	−0.416	2.242	0.978	−0.68	0.008	−0.644	0.082	0.645	1.852	0.841	0.18	−1.02	0.673	0.613
	CD4/CD8	0.108	−0.139	0.2	− 0.125	−0.185	0.242	0.126	0.717**	0.224	0.153	0.394	−0.366	−0.488**	− 0.372	−0.613	0.12
	VL(log)	0.404	0.445	0.164	0.201	−0.061	0.379	0.228	0.533**	0.579*	0.211	0.376	0.059	0.242	0.124	0.22	−0.083
	DI	0.547	−0.64	−0.088	2.21	0.848	−0.782	0.082	−0.672	−0.143	0.276	1.876	0.484	−0.167	−1.267	0.265	1.071
Cognitive Status	VF	−0.256	−0.333	− 0.194	−0.411	− 0.507**	−0.67**	− 0.502	0.196	− 0.671**	0.059	− 0.16	0.396	− 0.057	0.102	0.126	−0.437
	A/WM	0.237	0.238	−0.219	0.474	0.147	0.619**	0.461	0.201	0.566**	0.091	0.062	−0.078	0.245	−0.243	− 0.219	0.115
	EF	−0.278	−0.406*	− 0.385*	−0.305	0.045	−0.227	− 0.183	−0.394*	0.099	−0.406	− 0.251	−0.249	− 0.408*	−0.339*	− 0.24	0.394
	M(LDR)	0.011	−0.028	0.334	−0.148	−0.272	− 0.307	−0.132	0.245	0.079	0.067	0.134	−0.038	−0.272	− 0.074	0.116	0.068
	SIP	0.589**	0.382*	0.178	−0.049	0.206	0.136	0.176	0.631**	0.544**	0.082	−0.125	0.435	0.17	0.529**	0.362	0.123
	FM	−0.329	−0.447*	− 0.241	−0.179	− 0.268	−0.622**	− 0.441	0.081	− 0.232	−0.281	− 0.469	0.026	− 0.172	0.143	0.092	−0.406

The regression coefficients and significance results were calculated by the multiple linear analysis method. AD axial diffusivity, GCC genu of corpus callosum, *BCC* body of corpus callosum, *SCC* splenium of corpus callosum, *ACR* anterior corona radiata, *SCR* superior corona radiata, *L* left, *R* right, *ALIC* anterior limb of internal capsule, *EC* external capsule, *RIC* retrolenticular part of internal capsule, *PCR* posterior corona radiate, *PTR* posterior thalamic radiation, *SS* sagittal stratum, *SLF* superior longitudinal fasciculus, *VL* viral load, *DI* duration of infection, *VF* Verbal Fluency, *A/WM* Attention/Working Memory, *EF* Executive Functioning, *SIP* Speed of Information Processing, *MS* Motor Skills, * P < 0.1, ** P < 0.05

### Correlations between DTI metrics and cognitive performance for HIV-infected patients

**Fig. 3:**
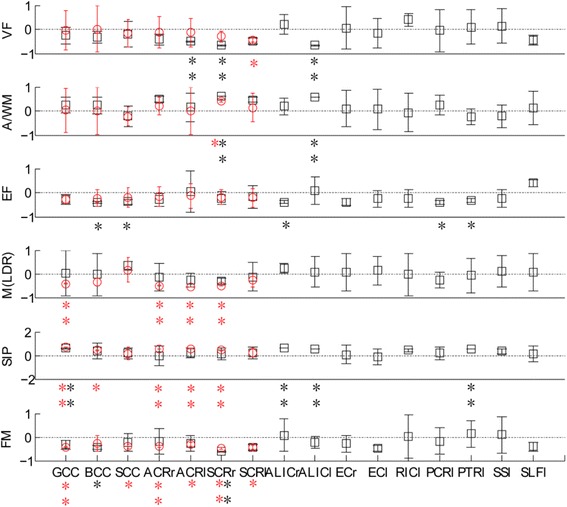
Regression coefficients and significance for white matter alterations and cognitive performance. Verbal fluency scores were negatively correlated with AD values in the left ACR, ALIC and right SCR. Attention/working memory scores were positively correlated with AD values in the left ALIC and right SCR. Memory (learning and delayed recall) test scores were negatively correlated with MD values in the GCC, ACR (bilateral) and SCR (right). A positive correlation was observed between speed of information processing scores and MD values in the GCC, ACR (bilateral) and SCR (right), AD values in the GCC, ALIC (bilateral) and PTR (left). Fine motor scores were negatively correlated with MD values in the GCC, ACR (right) and SCR (right), AD values in the SCR (right) Note: bar: P value; *: p < 0.1; **: p < 0.05; red circle: regression coefficients of MD; black square: regression coefficients of AD. MD, mean diffusivity; AD, axial diffusivity; GCC, genu of corpus callosum; BCC, body of corpus callosum; SCC, splenium of corpus callosum; ACR, anterior corona radiata; SCR, superior corona radiata; ALIC, anterior limb of internal capsule; EC, external capsule; RIC, retrolenticular part of internal capsule; PCR, posterior corona radiata; PTR, posterior thalamic radiation; SS, sagittal stratum; SLF, superior longitudinal fasciculus. l, left; r, right; VF, Verbal Fluency; A/WM, Attention/ Working Memory; EF, Executive Functioning; M(LDR), memory (learning and delayed recall); SIP, Speed of Information Processing; FM, fine motor.

Reduced cognitive scores were significantly correlated with either increased MD or AD in multiple white matter structures (Fig. 3, Table 3 and Table 4). Verbal fluency scores were negatively correlated with AD values in the left anterior corona radiata, anterior limb of the internal capsule and right superior corona radiata. Attention/working memory scores were positively correlated with AD values in the left anterior limb of the internal capsule and right superior corona radiate. Memory (learning and delayed recall) test scores were negatively correlated with MD values in the genu of the corpus callosum, anterior corona radiata (bilateral) and superior corona radiata (right). A positive correlation was observed between the speed of information processing scores and MD values in the genu of the corpus callosum, anterior corona radiata (bilateral) and superior corona radiata (right), AD values in the genu of the corpus callosum, anterior limb of the internal capsule (bilateral) and posterior thalamic radiation (left). Fine motor scores were negatively correlated with MD values in the genu of the corpus callosum, anterior and superior corona radiata (right), and AD values in the superior corona radiata (right).

## Discussion

This study not only supports but also further extends previous DTI findings in neuroasymptomatic HIV-positive individuals. One purpose of this study is to explore the microstructure changes of WM in treatment-naïve ANI patients employing the DTI-TBSS method. Compared with healthy controls, ANI patients exhibited significantly increased MD and AD in the corpus callosum and anterior and superior corona radiate. The corpus callosum and corona radiata were distributed around the lateral ventricle. The anterior limb of internal capsule, external capsule, retrolenticular part of the internal capsule, posterior corona radiata, posterior thalamic radiation, sagittal stratum, and superior longitudinal fasciculus presented significantly increased AD, and we found that most of them were also close to the ventricles. It is speculated that HIV-associated WM involvement is selective rather than random. Periventricular WM, especially the corpus callosum and corona radiata, are more vulnerable to viral invasion and neuroinflammation in early HIV infection in adults, which are consistent with previous studies [6–8, 32, 33]. Ragin et al. even found a loss of WM integrity in the corpus callosum within 100 days of HIV infection [33]. It is not clear why these regions were vulnerable to viral invasion and neuroinflammation. One possible explanation is that the choroid plexus is the blood-cerebrospinal fluid (CSF) barrier, and HIV can accumulate in the CSF when it is destroyed. Studies have shown that HIV can infiltrate the CSF as early as 8 days after exposure [34], and CSF serves as a proxy for the brain parenchyma and the reservoir for monocytes linked to HIV neuropathogenesis [35]. Early neuroinvasion was identified by measurable markers of CSF inflammation [34], and the WM tracts around the ventricle might be affected gradually. The interpretation of these results requires caution, and future investigation will be needed to better characterize them.

WM tracts with increased AD are more extensive than those of increased MD. The overlap and differences of the two DTI parameters (MD and AD) in significantly altered cerebral regions reflect differences in the nature and degree of WM injury. Increased MD indicates an increase of the water molecules’ diffusion speed, which is caused by cell degeneration and a decrease of membrane density. Increased MD may reflect inflammation or increased glial activation, a measure of neuronal injury. Increased AD is a marker of axonal chronic damage [7, 36]. Increased RD is associated with the destruction of myelin integrity and is used as a marker for demyelination [16, 17, 37]. RD abnormalities were not found in the current ANI study. It was concluded that MD changes in ANI subjects were mainly attributable to increased AD, suggesting chronic axonal injury rather than the disruption integrity of myelin in early HIV infection. A similar study found elevated CSF neurofilament light chain concentration and its correlation with MRS-based metabolites in primary HIV infection [32], and the neurofilament light chain is a sensitive marker of axonal injury. These findings demonstrate that axonopathy may exist before myelin injury, and this may be a novel observation. However, whether can axonal injury trigger demyelination (inside-out) is not yet clear. In recent DTI studies on ultra-early HIV infection, the authors focused only on FA and MD, and AD and RD were not calculated [23, 33]. AD and RD are also important, as they provide information on the nature of the WM microstructure alterations observed in HIV patients. Researching on multiple metrics of DTI may help us to comprehend the pathophysiology of HIV-related WM injury. The relationship of axonal and myelin injury needs to be better characterized in future HAND pathology studies.

FA abnormalities were also not found in ANI patients, which implies that RD maybe the predominant factor that contributes to decreased FA. Similar findings also have been reported, which showed that FA changes were attributable to increased RD [14, 38]. In addition, several DTI studies noted that significant alterations in FA were found mainly in cognitively impaired HIV-infected patients [7, 39]. Thus, MD may be a more sensitive biomarker than FA in evaluating WM injury in early HIV infection.

Our previous DTI study in early HIV infection showed significant differences in MD, AD, and RD values between a therapy naïve HIV+ group and healthy control group [29]. One resemblance between the two studies is the distribution of white matter abnormalities. Another similarity is that white matter abnormalities are all reflected in the changes of MD and AD values, rather than FA values. The difference is that the previous study has a wider range of WM injury. Additionally, changes of RD values can be seen in a few WM tracts (genu of corpus callosum and superior corona radiate), which indicates myelin damage. A possible reason is that HIV-infected patients in the previous study were classified into ADC stage 0 according to the MSK classification. While MSK is a decent scale to globally express the state of cognitive functioning, it is not very sensitive to changes in less affected patients. The Frascati scale used in the current study may be more sensitive to identify and classify individuals with subclinical impairment. More detailed neuropsychological assessment for earlier HIV-infected patients is the novel element of the current study relative to the previous study. Corrêa et al. found that HIV patients with planning deficits had significantly decreased FA, increased MD and RD values, predominantly in frontal lobes, genu and splenium of the corpus callosum, and much less widespread abnormalities were seen in the AD values compared with normal controls. HIV+ patients with planning deficits also had significantly decreased FA values and increased MD and RD values in some white matter regions compared to those without planning deficits [40]. No significant abnormalities AD values were seen between the two groups. These results indicated that RD abnormal values predominated in the areas of decreased FA compared to AD values, suggesting that demyelination could play a role in the physiopathology of HIV-related WM injury, which is not completely consistent with our results. The possible reason for the difference between the two results was that participants in the previous article all received ART, and with longer known infection. Antiretroviral drugs may be injurious to brain cell elements. The influence of treatment on brain structure and function are less clear [41]. HIV+ patients on low CNS penetration ART had a significantly greater fMRI response amplitude compared to the HIV+ patients on high CNS penetration ART or normal controls [42]. To the best of our knowledge, no studies have detected the effects of ART regimen CNS penetration effectiveness on WM microstructure. Effects of treatment should be explored in future studies.

The MD values in the right anterior corona radiata and AD values in the left posterior corona radiata have a significantly negative correlation with CD4^+^/CD8^+^ ratios, and the regression coefficient is 0.437 and 0.488, respectively; in other words, WM microstructure changes (43.7% in the anterior corona radiata, 48.8% in the posterior corona radiate) can be influenced by CD4^+^/CD8^+^ ratios. The abnormality of AD values was related to axonal chronic injury. Clinically, the lower CD4^+^/CD8^+^ ratios were related to immunosenescence [43]. This might imply that immunosenescence among the ANI patients would accelerate the axonal chronic injury in the corona radiata, and the lower CD4^+^/CD8^+^ ratio might be an important predictor of WM injury in the corona radiata. Furthermore, we found that plasma viral load remained independently associated with AD values in the right anterior limb of the internal capsule, and the regression coefficient was 0.533. The higher the plasma viral load, the higher are the AD values in the right anterior limb of the internal capsule. The higher plasma viral load represents the activity and replication of HIV in the human internal environment. The positive correlation indicates that the WM microstructure in the anterior limb of the internal capsule was susceptible to viral replication in HIV infection. The current findings were not fully consistent with previous studies [7, 11, 22–25], and the results of previous studies also varied. This may be due to the difference and heterogeneity in participants with diverse treatments, sample size, cognitive status, and disease durations. Further studies are needed to resolve this incongruity and reliability.

Associations between WM microstructure alterations and cognitive impairment were observed in the current study. Several other studies have reported that WM changes were related to HIV-associated cognitive difficulties [44–46]. WM microstructure changes in the anterior and superior corona radiata and anterior limb of the internal capsule were significantly correlated with poorer verbal fluency. WM damage in the genu of the corpus callosum and anterior and superior corona radiata were significantly correlated with poorer memory (learning and delayed recall) and slower fine motor speed. The corona radiata is the radiated projection fibre connecting the internal capsule to the cerebral cortex. The corona radiata and internal capsule are important WM nodes that promote the transfer of sensorimotor information between the brain stem, thalamus and frontostriatal circuit [47]. The anterior corona radiate connects the anterior and medial nuclei of the thalamus to the prefrontal cortex. The superior corona radiate involves corticospinal tracts and the posterior frontal part of the anterior thalamic radiation [7]. The corpus callosum is the largest and most prominent WM tract, which is responsible for the communication of interhemispheric information. The genu of the corpus callosum contains the posterior frontal part of callosal fibres [7]. The corpus callosum and corona radiate are pivotal in extensive cognitive function, such as verbal fluency, attention, memory, psychomotor speed and executive functioning. The significant correlation between neurocognitive performance and MD and AD values from multiple WM microstructures suggests that WM abnormalities have functional consequences. HIV-related cognitive impairment may be associated with cortical and subcortical track loss caused by WM fibre bundle damage, and the WM microstructure may serve as an indicator to objectively predict cognitive deficits and progression. However, a multivariate model also showed that the WM microstructure alterations in the superior corona radiate and anterior limb of the internal capsule better predicted sustained attention/working memory. WM injury in the genu of the corpus callosum, anterior and superior corona radiata, anterior limb of the internal capsule and posterior thalamic radiation were significantly correlated with faster speed of information processing. A potential explanation for this may be some sort of compensatory mechanism, and it needs to be further verified in future multimodal studies (DTI combined functional MRI).

There were several limitations in the current study. First, the study was limited to a small sample size. A larger sample size would be more helpful to improve the power of the statistical analysis. Second, the participants were almost exclusively male, which may prevent the generalization of these results to HIV-infected women. Certainly, given that the gender gap is narrowing with rates of infection increasing in women, we are trying our best to extend our studies to include female patients in the future. Third, a cognitively intact HIV-positive group will be studied in further work. Fourth, a longitudinal follow-up study is imperative to observe the dynamic changes of WM after ART.

## Conclusions

The observations of the current study strengthen the possibility that HIV-infected individuals at the ANI stage have underlying WM fibre abnormalities, which could be measured by increased MD, and the pathogenesis of this damage is likely to be predominantly the axonal chronic injury associated with increased AD. DTI has the potential to promote a better understanding of the pathogenesis of brain WM changes. Specific brain regions around the ventricle, especially the corpus callosum and corona radiata, are susceptible to be involved. Relationship exists between WM damage, HIV-related clinical factors, and cognitive status. HIV patients with a history of advanced immune suppression and higher viral load may be at high risk of WM injury. WM damage and disconnection to the cortex probably contribute to cognitive impairments.

## Abbreviations

AD: Axial diffusivity
ADC: AIDS Dementia Complex
ANI: Asymptomatic neurocognitive impairment
ART: Antiretroviral therapy
CNS: Central nervous system
CSF: Cerebrospinal fluid
DTI: Diffusion tensor imaging
FLAIR: Fluid attenuated inversion recovery
HAND: HIV-associated neurocognitive disorders
MD: Mean diffusivity
MRI: Magnetic resonance imaging
MSK: Memorial Sloan Kettering
RD: Radial diffusivity
TBSS: Tract-based spatial statistics
WM: White matter
